# Fragment of tegument protein pp65 of human cytomegalovirus induces autoantibodies in BALB/c mice

**DOI:** 10.1186/ar3481

**Published:** 2011-10-11

**Authors:** Ao-Ho Hsieh, Yí-Jyun Jhou, Chung-Ting Liang, Mingi Chang, Shih-Lien Wang

**Affiliations:** 1 Institute of Medical Science, Tzu-Chi University, No. 701, Sec. 3, Zhongyang Rd., Hualien City, Hualien County 970, Taiwan; 2 Institute of Microbiology Immunology and Biochemistry, Tzu-Chi University, No. 701, Sec. 3, Zhongyang Rd., Hualien City, Hualien County 970, Taiwan; 3 The National Laboratory Animal Center (NLAC), No. 128, Sec. 2, Academia Rd., Nangang Dist., Taipei City 115, Taiwan; 4 Development Center of Biotechnology, No.101, Ln. 169, Kangning St., Xizhi Dist, New Taipei City 221, Taiwan

## Abstract

**Introduction:**

Human cytomegalovirus (HCMV) infection has been implicated in the development of autoimmunity, including systemic lupus erythematosus (SLE). Previously we reported that HCMV phosphoprotein 65 (pp65) could induce early onset of autoantibody and glomerulonephritis on lupus-prone NZB/W mice. This study further examined whether the B cell epitope(s) in pp65 is able to drive the development of autoantibody.

**Methods:**

Sera from SLE patients or HCMVpp65-immunized mice were analyzed for anti-nuclear antibody by immunoblotting, enzyme-linked immunosorbent assay (ELISA), immunofluorescent stain and *Crithidia luciliae *stain. The deposition of immunoglobulin to the kidney was also examined by immunofluorescent stain. The interactions between pp65 sub-fragment to cellular proteins were revealed by yeast two-hybrid analyses.

**Results:**

Our results showed that most SLE patients possessed antibodies to the C-terminal half of the HCMVpp65 antigen. Of these positive sera, 73% were also positive to the pp65_336-439 _sub-fragment. The immunization of pp65_336-439 _induced formation of multiple anti-nuclear antibodies, including anti-chromatin, anti-centriole, anti-mitotic spindle type I/II (MSA I/II) and a significant elevation of anti-double-stranded DNA (anti-dsDNA) antibodies on BALB/c mice. Yeast two-hybrid analyses revealed the binding of pp65_336-439 _sub-fragment to cellular proteins. Immunoglobulin deposition on glomeruli was also detected on pp65_336-439_-immunized mice.

**Conclusions:**

Our data suggested that HCMVpp65_336-439 _sub-fragment may induce cross-reactive antibodies to several nuclear antigens, which could contribute to the development of autoimmunity in genetic-suspected individuals.

## Introduction

The Epstein-Barr virus (EBV)-infection-induced systemic lupus erythematosus (SLE)-specific autoantibody is one of the best examples for cross-reactive antibody mediated autoimmunity [1]. In those studies, autoantibodies to Smith antigen B/B' (SmB/B') and clinical symptoms that resemble SLE were induced by normal strains of mice following immunization of octapeptide (PPPGRRP) [2]. The amino acid sequence is not a reliable indicator to predict cross-reactivity because antibodies to amino acid 52 to 72 of Epstein-Barr virus nuclear antigen 1 (EBNA-1_52-72_) also cross-reacted to amino acid 169 to 180 of Ro antigen (Ro_169-180_) disregard significant differences of both sequences [3].

HCMV belongs to the *Betaherpesvirinae *family and is an opportunistic pathogen that could cause severe clinical consequences in individuals with impaired immune systems [4]. Specific activation of both viral-specific and auto-reactive T-cells during infection has been shown to accelerate the development of type I diabetes [5,6]. HCMV-infection-induced Ro60 antigen expression on the cell surface and elevated anti-phospholipid antibody has been reported [7,8]. In addition, a higher prevalence of autoantibody to U1 small nuclear ribonucleoprotein (U1 snRNP) in SLE patients and animals are associated with HCMV infection or immunization, respectively [9,10]. The tegument phosphoprotein 65 (pp65, UL83) of HCMV is the most abundant phosphoprotein on the virion and an immunodominant target to both CD4^+ ^and CD8^+ ^T cells [11,12]. Two T-cell dominant regions, pp65_303-388 _and pp65_477-561_, located on the C-terminus of pp65, have been reported and at least 28 CTL epitopes were verified within the CMVpp65 [13,14].

It has been demonstrated that in addition to activating T-cells, immunization of pp65 encoded plasmid could induce early onset of autoantibody activity and glomerulonephritis on lupus-prone animals [15]. The anti-pp65 antibody activity is not a common feature of healthy individuals, only 11.11% normal sera (sera from healthy donors) possess antibodies to pp65 antigen [15]. Immunization of pp65 antigen or its fragments in Freund's adjuvant to BALB/c mice only elicited anti-pp65 activity for a limited time [15]. The C3d is a degraded peptide of the third complement complex protein and ligand to complement receptor 2 (CR2/CD21). Because of its CD21 binding property, C3d has been used as an adjuvant to enhance the immunization efficiency or to activate anergic B cells [16-18]. Here, we reported that immunization of pp65_336-439 _with C3d as adjuvant to BALB/c mice induced diverse nuclear-targeting autoantibodies and immunoglobulin deposition on glomeruli. Moreover, pp65_336-439 _induced immunity cross-reacts to multiple cellular proteins suggesting that immune responses to pp65_336-439 _may instigate autoimmunity.

## Materials and methods

### Human sera

This study involving human subjects was approved by the Tzu-Chi University, National Science Committee and the National Blood Center or Taichung Veteran Hospital Review Boards and approved by the Committee of Ethics in Tzu-Chi University [15]. A selected portion of patients' sera were removed from this study subsequently due to restriction from Institutional Review Boards. All subjects in this study gave their informed consents. Patients were classified based on the classification criteria of the American College of Rheumatology as SLE (*n *= 61), rheumatoid arthritis (RA, *n *= 50), Sjögren's syndrome (SS, *n *= 13) and systemic sclerosis (SSc, *n *= 20). Normal sera (*n *= 45) were collected from qualified, sex- and age-matched adult blood donors.

### Mice

Normal six- to eight-week-old female BALB/c mice were purchased from the National Laboratory Animal Center (NLAC), Taipei, Taiwan. Animals were housed in a pathogen-free facility with an independent ventilation cage system at the Laboratory Animal Center of Tzu-Chi University, Hualien, Taiwan. All animal experiments were approved by Tzu-Chi University Animal Experimental Ethics Committee (reference number 94-A-06).

### Constructions and expression plasmids

The pp65_1-167_, pp65_167-336 _and pp65_336-561 _sequences are amplified using the following primer pair sequences, respectively listed in Table 1. The sequences were designed using the published nucleotide sequence of pp65 (strain: AD-169, GenBank: FJ527563). The fragments of pp65_1-167_, pp65_167-336_, and pp65_336-561 _were prepared from PCR and digested by restrictive enzymes, and then ligated into pET30. The pp65_336-379_, pp65_379-455 _and pp65_455-561 _fragments were digested from pp65_336-561 _to form 132-bp (*Bam*HI/*Hin*dIII), 231-bp (*Hin*dIII/*Not*I) and 321-bp (*Not*I/*Xho*I) fragments, respectively. The pp65_336-422_, pp65_336-439 _and pp65_336-448 _encoding sequences were amplified from a pp65_336-561 _clone using both upstream and downstream primers (Table 1). The pp65 sub-fragments mentioned above were cloned and inserted into pET30. The murine C3d encoding sequence (GenBank: DQ408205) was PCR amplified with C3d primers and ligated into pET32 (Table 1). For yeast two-hybrid analysis, PCR product of pp65_336-439 _was cloned into pAS-1 plasmid to create a pAS-1-pp65_336-439_-binding domain (BD) plasmid.

**Table 1:** Primers sets for the truncation of HCMVpp65 antigen constructions

Clones	Sequences		Vectors
	**Forward (5- > 3)**	**Reverse (5- > 3)**	

pp65_1-167_	ATG GAT CCA TGG AGT CGC GCG GTC GCC G	CCG GAA TTC CAG TCC CGA GAC CGT GAG GAC CGT	pET30
pp65_167-336_	CGC GGA TCC TGG ACG CGT CAG CAG ACC CA	CGC GGA TCC CTC GCG TAT GGC TTG TAC CT	pET30
pp65_336-561_	CGC GGA TCC ACC GTG GAA CTG CGT CAG TA	TAG GAT CCA CCT CGG TGC TTT TTG GGC G	pET30
pp65_336-448_	CGC GGA TCC ACC GTG GAA CTG CGT CAG TA	CGC CTC GAG CGA CGT GCA CGC CGT CGC	pET30
pp65_336-439_	CGC GGA TCC ACC GTG GAA CTG CGT CAG TA	CGC CTC GAG TGA TTT GCG TTT GCG GCC	pET30
pp65_336-422_	CGC GGA TCC ACC GTG GAA CTG CGT CAG TA	CGC CTC GAG GCC GGTGAC GCG GGG CGT	pET30
murine C3d	CGC GAT ATC ACC CCC GCA GGC TGT GGG GAA C 3'	CGC GGA TCC GGA TCC GCT ACG GCT GGG GAG	pET32
pp65_336-439_	CGC GGA TCC ACC GTG GAA CTG CGT CAG TA	CGC CTC GAG TGA TTT GCG TTT GCG GCC	pAS-1

The underlined sequences mean the usage of restrictive enzyme

### Antigen preparation, biotinylation and streptavidin conjugation

Recombinant proteins were over-expressed in *E. coli *with 1 mM isopropyl β-D-thiogalactoside (IPTG, Sigma-Aldrich, St. Louis, MO, USA) induction and purified by nickel affinity column. The C3d biotinylation and streptavidin (SA) conjugation (Pierce, Thermo Scientific, Rockford, IL, USA) were performed by the manufacturers' instructions. In brief, maleimide-activated streptavidin (Pierce) was conjugated with proteins containing reduced disulfide bonds from a disulfide reducing gel (Pierce) and mixed with biotinylated C3d to form the protein-SA-C3d tetramer, including pp65_1-167_, pp65_336-439 _and SA-C3d only. Tetramers were generated and prepared for immunization within four hours.

### Immunization and sera collection

A total of 35 six- to eight-week-old female BALB/c mice were randomly separated into groups of pp65_1-167_-C3d (*n *= 11), pp65_336-439_-C3d (*n *= 17), SA-C3d (*n *= 5) and PBS (*n *= 2). Mice were inoculated intraperitoneally with 50 μg pp65_336-439_-C3d or pp65_1-167_-C3d, or SA-C3d in complete Freund's adjuvant (Complete Freund's Adjuvant, Sigma-Aldrich) or phosphate-buffered saline (PBS, 3.2 mM Na_2_HPO_4_, 0.5 mM KH_2_PO_4_, 1.3 mM KCl, 135 mM NaCl, pH 7.4). Boosting was performed with antigens in incomplete Freund's adjuvant (Incomplete Freund's Adjuvant, Sigma-Aldrich) three times in three weeks. Mice were bled via the retro-orbital vein one day prior to each assay and at two-week intervals. Unused sera were stored at -20°C and the diluted sera for use were kept at 4°C.

### Immunoblotting and enzyme-linked immunosorbent assay

Immunoblotting was performed as previously described [15]. In brief, 1 × 10^8 ^cultured HeLa cells or 2 μg purified HCMV were prepared, homogenized and separated by 12% sodium dodecyl sulfate polyacrylamide gel electrophoresis (SDS-PAGE/slab gel format). Separated proteins were transferred to nitrocellulose paper and blocked by 5% skim milk then analyzed with mice or human sera at dilutions of 1:500 or 1:1,000 in PBS. The antibody reactivity was detected by horseradish peroxidase (HRP) conjugated secondary antibody (Jackson ImmunoResearch Laboratories, West Grove, PA, USA) and chemiluminescent detection agents (Perkin Elmer, Norwalk, CT, USA).

ELISA was performed as previously described [15]. In brief, for the anti-dsDNA antibody assay, 1 μg/well of purified calf thymus dsDNA (Sigma-Aldrich) in ddH_2_O was coated to a microtiter plate (Corning, Lowell, MA, USA). After blocking with 5% skim milk, mice or human sera at 1:100 and 1:1,000 dilutions in PBS, respectively, were added and incubated at room temperature (RT) for two hours. At the end of incubation, the plate was washed and bound antibodies were detected by HRP conjugated secondary antibodies at dilutions of 1:10,000 (for anti-dsDNA IgG) or 1:2,000 (for anti-dsDNA IgG subtypes, Bethyl Laboratories, Montgomery, TX, USA, purified HCMV or 1 μg/well of HeLa lysate in PBS were coated on a microtiter plate at 4°C overnight [15]. After the plate was skim-milk-blocked, mouse or human sera were added at dilutions of 1:500 or 1:1,000, respectively, in PBS and incubated at 4°C for two hours. The bound antibodies were detected by a secondary antibody at dilutions of 1:10,000 at 4°C for two hours. The o-phenylenediamine dihydrochloride (OPD, Sigma-Aldrich) was used as the substrate and HRP activity was read at 450 nm with a micro-ELISA reader (DYNEX MRX II).

For the detection of protein-to-protein interaction between pp65_336-439 _and HeLa proteins, whole HeLa extract (1 × 10^8 ^cultured HeLa cells) was separated by 12% SDS-PAGE/slab-gel and transferred onto nitrocellulose paper. Before the experiment, the blot was cut into strips, skim-milk-blocked and then incubated with either pp65_336-439 _or pp65_1-167 _His-tag fusion protein at concentrations of 20, 10, 5, 2.5, 1.25, 0.625 mg/ml or at concentrations of 20, 10, 5, 2.5 mg/ml, respectively for one hour. The pp65_336-439 _or pp65_1-167 _bound HeLa proteins were detected by 10,000X diluted HRP-conjugated mouse anti-His tag IgG (Serotec, Raleigh, NC, USA) after one hour of incubation. The reactions were visualized by chemiluminescent detection agents.

### Immunofluorescence

Mouse sera were tested for anti-nuclear antibodies (ANAs) at a dilution of 1:100 in PBS by a standard anti-nuclear antibody (ANA) test (Binding site). The reactivity of anti-dsDNA antibody was tested by immunofluorescent stain using the *Crithidia luciliae *test (binding site) with mice sera at a dilution of 1:40 in PBS, as suggested by the manufacturer. In brief, 25 μl of diluted mice sera were incubated with slide-coated HEp-2 or *Crithidia luciliae *for 20 minutes in humid chamber at RT. HEp-2 or *Crithidia luciliae *slides were washed three times in PBS at RT for 10 minutes each. The bound antibodies were detected by 100X diluted Fluorescein isothiocyanate (FITC)-conjugated anti-mouse IgG (Jackson ImmunoResearch Laboratories) for 20 minutes in a humid chamber at RT in the dark. For nuclear visualization, HEp-2 slide was incubated in 25 μl of DAPI (0.5 μl/ml, Sigma-Aldrich) at RT for two minutes in the dark. At the end of staining, slides were washed (PBS) and mounted (mounting medium) for investigation by Nikon E800 fluorescence microscopy (Nikon, Tokyo, JP).

For an immunofluorescent stain on glomerulus, kidneys were removed from mice, immediately placed in the OCT gel and frozen at -80°C for 24 hours. The 5-mm-thick frozen sections were stained with FITC-conjugated anti-mouse IgG at a 1:100 dilution in PBS for 20 minutes in a humid chamber at RT in the dark. After PBS washing, coverslips with mounting medium on tissue slides were prepared for investigation by Nikon E800 fluorescence microscopy.

### Antibody purification

Moderated Cyanogen bromide (CnBr) powder (Sigma-Aldrich) was activated as described by the manufacturer. In brief, purified and sonicator-homogenized HCMV virions were dissolved in a coupling buffer (0.1 M NaHCO_3_, 0.5 M NaCl, pH 8.3) with activated CnBr gel at 4°C overnight. The free active groups on CnBr were deactivated by 0.1 M Tris-HCl (pH 8.0) at RT for two hours. After deactivation, CnBr gel was washed with alternating buffer (0.1 M NaAc, 0.5 M NaCl, pH 4.0 and 0.1 M Tris-HCl, 0.5 M NaCl, pH 8.0) twice and washed with 10 ml PBS once. For purification, 200 μl of pooled pp65_336-439 _or pp65_1-167 _mouse sera in 10 ml PBS were added to HCMV-CnBr gel and rolled at 4°C overnight. The unbound portion of sera, flow through, was collected and concentrated as a negative control, while bound antibodies were eluted by 1 ml of 0.1 M glycine (pH 2.0) [19]. The eluted samples were neutralized immediately with a 30 μl of neutralizing buffer (1 M Tris-HCl, 2 M NaCl, pH 8.8).

### Yeast two-hybrid screening

The Matchmaker yeast two-hybrid screening system (Clontech. Mountain View, CA, USA) was used to identify the proteins that were able to interact with pp65_336-439 _peptide. In this system, yeast two-hybrid library screening using yeast mating was performed as modified from the manufacturer's manual. The DNA fragment encoding pp65_336-439 _was cloned into the Gal4BD (DNA-binding domain of the transcription factor Gal4) vector pGBT-7 and the resulting plasmid was designated as BD-pp65_336-439_. The BD-pp65_336-439 _plasmid was transformed into the yeast strain AH109 (MATa) for screening the yeast library strain (Clontech Co.), which is the yeast strain Y187 (Matα) transformed with the AD-cDNA plasmid, HeLa cDNA cloned into the AD (activation domain of Gal4) vector pGAD-T7. The AH109 cells bearing BD-pp65_336-439 _were cultured in the synthetic dextrose medium lacking tryptophan at 30°C until O.D._600 _was approximately 0.8. The AH109 cells bearing BD-pp65_336-439 _were then collected and mated with the yeast library strain in 2X YPDA medium (1% Bacto yeast extract, 2% Bacto peptone, 2% Dextrose, 4% Adenine hemisulfate) at 30°C. After mating, cells were screened on the synthetic dextrose solid medium lacking leucine, tryptophan and histidine (SD/-L/-W/-H) to assay expression of reporter gene *HIS3 *at 30°C. The screened colonies were further screened on the synthetic dextrose solid medium lacking leucine, tryptophan, histidine and adenine (SD/-L/-W/-H/-A) to assay expression of reporter genes *HIS3 *and *ADE2 *at 30°C. The AD-cDNA plasmid was isolated from the screened colony grown on the SD/-L/-W/-H/-A solid medium and transformed into *E. coli *for amplification. To further confirm the interaction between pp65_336-439 _peptide and the cDNA-encoding protein, both BD-pp65_336-439 _plasmid and the purified AD-cDNA plasmid were transformed into yeast strain YRG2 (Stratagene, La Jolla, CA, USA) and tested on the SD/-L/-W/-H solid medium containing 15 mM 3-aminotriazole to assay for the expression of the reporter gene *HIS3 *at 30°C. The purified AD-cDNA plasmid was then sequenced after confirmation.

### Statistical analysis

Statistical methodology for differences of titer and prevalence in test results was analyzed by GraphPad Prism (GraphPad Software Inc. La Jolla, CA, USA) and using the Student *t*-test and Fisher's two-tailed exact test, respectively. Results with a *P-*value of < 0.05 were considered to be significant.

## Results

### The pp65_336-439 _sub-fragment of HCMV contains a B cell epitope(s) targeted by IgG from SLE patients

**Figure 1:**
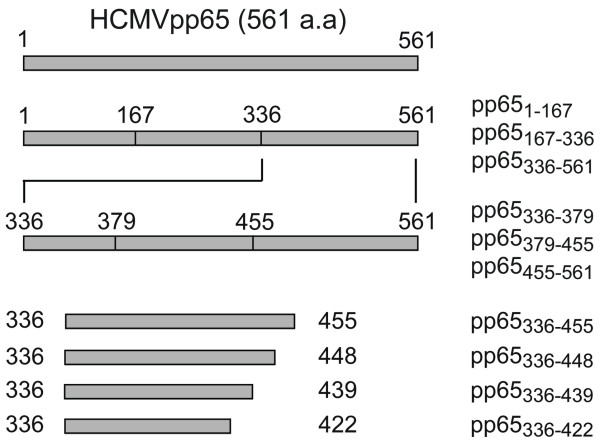
**Schematic representation of truncated HCMVpp65 His-tag fusion proteins (Swiss-Prot**: P06725). The full-length of HCMVpp65 is given in the top figure. Below that, six pp65 sub-fragments, pp65_1-167_, pp65_167-336_, pp65_336-561_, pp65_336-379_, pp65_379-455_, pp65_455-561_, and four C terminal truncated peptides, pp65_336-422_, pp65_336-439_, pp65_336-448_, pp65_336-455_, are shown. The name of the plasmids which encoded pp65 sub-fragment proteins are given at the right. HCMVpp65, human cytomegalovirus phosphoprotein 65 (65 kD).

To verify the existence of B-cell epitope(s), HCMVpp65 tegument protein (pp65) was cloned, truncated and expressed as his-tagged fragments (pp65_1-167_, pp65_167-336 _and pp65_336-561_) that covered the entire antigen (Figure 1). Results showed that HCMV-seropositive SLE patients responded strongly to pp65_336-561 _(61%, 37/61) compared to pp65_1-167 _(7%, 4/61) or pp65_167-336 _(20%, 12/61). The elevated positive rate to pp65_336-561 _by SLE patients' sera was not found on either healthy or other disease controls (Table 2). In order to reveal the dominant epitope(s) within pp65_336-561_, pp65_336-561 _was sub-cloned into three fragments, expressed and re-screened (Figure 1). Of the original 37 pp65_336-561_-positive sera, 7 were removed from subsequent tests due to various availability issues. Of the rest of the 30 pp65_336-561_-positive sera, 22 were positive to pp65_379-455 _(73%, 22/30), 3 were positive to pp65_455-561 _(10%, 3/30) and 0 was positive to pp65_336-379 _(Table 2). Subsequently, pp65_336-448_, pp65_336-439 _and pp65_336-422 _fragments were created by partial deletion from the C-terminus of pp65_336-561 _(Figure 1). Of 22 pp65_379-455_-positive sera, 17 were positive to pp65_336-448 _(77%, 17/22), 16 were positive to pp65_336-439 _(73%, 16/22) and 9 were positive to pp65_336-422 _(41%, 9/22). The sero-reactivity to three fragments (pp65_336-422_, pp65_336-439 _or pp65_336-448_) by pp65_379-455 _positive sera was listed in Table 3.

**Table 2:** The prevalence of antibody to HCMVpp65 sub-fragments in patients with autoimmunity and healthy controls

	Antigens	SLE	SSc	SS	RA	Normal
Age (years)		11 to 77	48 to 79	14 to 79	21 to 81	31 to 73
Mean (years)		33.8	61.9	53.4	54.6	63.2
Total specimen		61	20	13	50	45
Female (%)		93.4	95	92.3	80	57.8
Responsiveness	pp65_1 to 167 _(%)	4/61 (7)	3/20 (15)	5/13 (38)	0/50 (0)	6/45 (13)
	pp65_167 to 336 _(%)	12/61 (20)	5/20 (25)	3/13 (23)	16/50 (32)	9/45 (20)
	pp65_336 to 561 _(%)	37/61 (61)	4/20 (20)	2/13 (15)	7/50 (14)	2/45 (4)
	pp65_336 to 379 _(%)	0/30 (0)				
	pp65_379 to 455 _(%)	22/30 (73)				
	pp65_455 to 561 _(%)	3/30 (10)				
	pp65_336 to 448 _(%)	17/22 (77)				
	pp65_336 to 439 _(%)	16/22 (73)				
	pp65_336 to 422 _(%)	9/22 (41)				

RA, rheumatoid arthritis; SS, Sjögren's syndrome; SSc, systemic sclerosis. For immunoblot and ELISA assay, detection of anti-pp65 sub-fragments antibody was tested with purified human cytomegalovirus (HCMV) virions, as described in materials and methods. The HCMV positivity is defined by mean + 3 s.e.m of normal control. The results are representative of triplicated experiments.

**Table 3:** The sero-reactivity to pp65 sub-fragments by pp65_379-45__5 _positive sera

Sero-reactivity to antigens			Patient number
pp65_336-422_	pp65_336-439 _	pp65_336-448_	*n *= 22
**+**	**+**	**+**	1, 4, 5, 9, 13, 17, 18, 19, 22
**-**	**+**	**+**	7, 10, 11, 12, 14, 16, 20
**+**	**-**	**+**	
**+**	**+**	**-**	
**-**	**-**	**+**	21
**-**	**+**	**-**	
**+**	**-**	**-**	
**-**	**-**	**-**	2, 3, 6, 8, 15

**+**, positive; **-**, negative

### Induction of anti-HCMV antibody and anti-HeLa protein antibody

**Figure 2:**
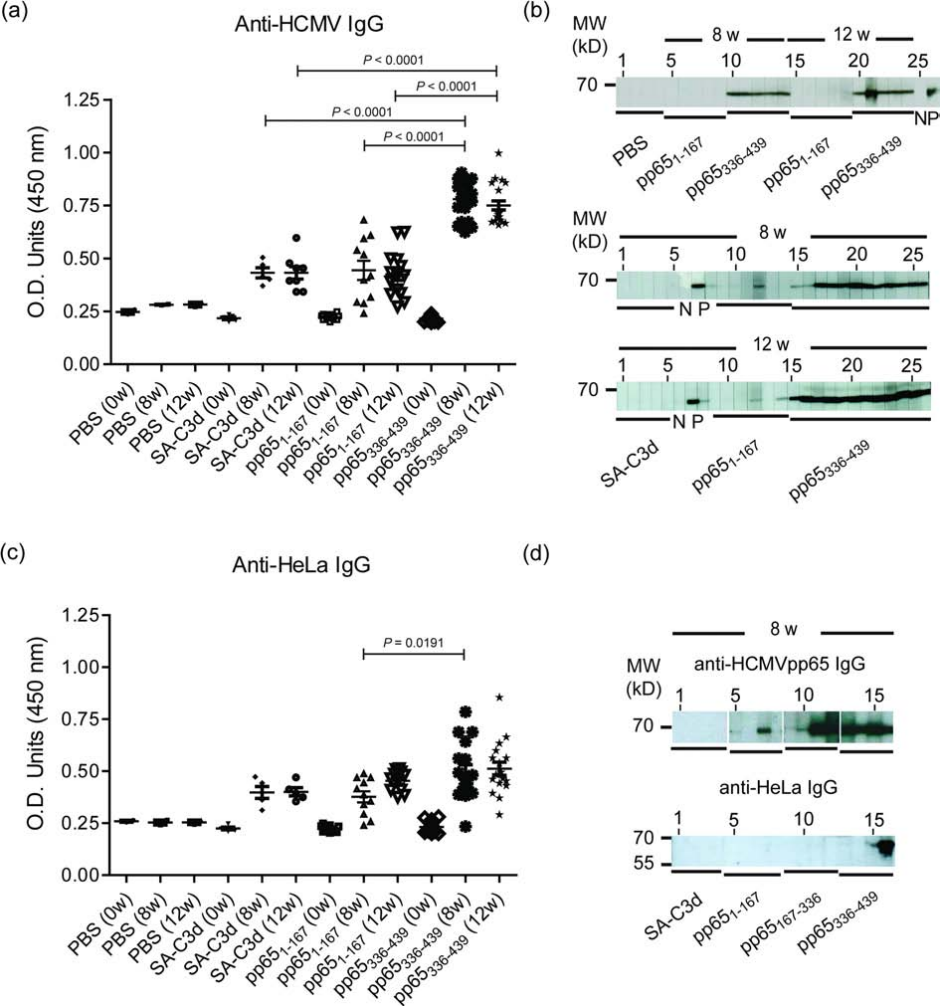
**Detection of anti-HCMVpp65 antibody by ELISA and immunoblot assay in immunized BALB/c mice**. The IgG against HCMVpp65 or HeLa extract from pp65_1-167 _(*n *= 11), pp65_336-439 _(*n *= 17), SA-C3d (*n *= 5) or PBS (*n *= 2) immunized mice. (**a**) ELISA assays for anti-HCMV reactivity against purified HCMV virions. Sera were 500X diluted and positivity was defined by mean + 3 s.e.m. of SA-C3d-immunized sera. O.D._450 _> 0.50 was considered to be positive. (**b**) Immunoblot analysis on anti-HCMV reactivity against purified HCMV virions. Sera were 500X diluted. Top panel: pp65_1-167 _(*n *= 5), pp65_336-439 _(*n *= 5) and PBS (*n *= 2). Lane 1 to 2 and 3 to 4, PBS-immunized sera at 8 and 12 weeks, lane 5 to 9 and 15 to 19, pp65 to _1-167_-immunized sera, lane 10 to 14 and 20 to 24, pp65_336-439_-immunized sera, lane 25, N: 1,000X diluted healthy control serum, lane 26, P: 1,000X diluted SLE patient's serum. Bottom panel: SA-C3d (*n *= 5), pp65_1-167 _(*n *= 6) and pp65_336-439 _(*n *= 12). Lane 1 to 5, SA-C3d-immunized sera, lane 6, N: 1,000X diluted healthy control serum, lane 7 to 8, P: 1,000X diluted SLE patients' sera, lane 9 to 14, pp65_1-167_-immunized sera, lane 15 to 26, pp65_336-439_-immunized sera. (**c**) ELISA assays for anti-HeLa reactivity against total HeLa lysate. Sera were 500X diluted. O.D._450 _> 0.48 was considered to be positive. (**d**) Immunoblot analysis with mouse sera at eight weeks post-immunization against HCMV and total HeLa lysate. Top panel: purified HCMV virion blot. Bottom panel: total HeLa lysate blot. Lane1 to 4, C3d-immunized sera, lane 5 to 8, pp65_1-167_-immunized sera, lane 9 to 12, pp65_167-336_-immunized sera, lane 13 to 16, pp65_336-439_-immunized sera. Molecular mass markers (kD) are shown on the left. MW: molecular weight. w: weeks of post-immunization. Graphs depict mean ± s.e.m. values. Unpaired Student *t *test was performed. Results with a *P-*value of < 0.05 were considered to be significant. These results are representative of triplicated experiments.

Although pp65 antigen immunized BALB/c animals possessed anti-pp65 antibodies and enhanced autoantibody activities, the titers were reduced in a few weeks after immunization [15]. To improve the immunogenicity of pp65, C3d was used as an adjuvant. The immunization results showed that pp65_336-439_-immunized mice gradually increased developed anti-HCMVpp65 IgG reactivity started at four weeks and continued to the end of the experiment (12 weeks post-immunization, Figure 2a, b). In contrast, the titer of anti-HCMVpp65 IgG was significantly less for pp65_1-167_-immunized mice (Figure 2a). The anti-HCMVpp65 IgG was not detected from either SA-C3d or PBS challenged mice (Figure 2b). Quasi-quantitative analysis showed that the titers of anti-HCMVpp65 IgG from pp65_336-439 _immunization was twice as much as sera from either pp65_1-167 _(pp65_336-439 _vs. pp65_1-167_, 0.78 ± 0.02 vs. 0.44 ± 0.05, *P *< 0.0001) or SA-C3d (pp65_336-439 _vs. pp65_1-167_, 0.78 ± 0.02 vs. 0.43 ± 0.02, *P *< 0.0001) immunized animals at eight weeks post-immunization (Figure 2a). The IgG reactivity to HCMV of pp65_1-167 _and SA-C3d was statistically insignificant.

In order to demonstrate that the immunization of pp65_336-439 _could lead to the development of cross-reactive autoantibodies, total HeLa lysate was prepared as the substrate for the detection of anti-HeLa antibodies (Figure 2c). Although immunization of pp65_336-439 _and pp65_1-167 _induced anti-HeLa IgG at 4 weeks and continued to 12 weeks post-immunization, pp65_336-439 _immunization exhibited significantly higher anti-HeLa IgG activity than pp65_1-167 _immunization (pp65_336-439 _vs. pp65_1-167_, 0.50 ± 0.03 vs. 0.38 ± 0.02, *P *= 0.0191) at 8 weeks post-immunization. To exclude the possibility of HCMV contamination, HeLa lysate were immunoblotted by pp65 sub-fragment immunized sera (Figure 2d). The results showed that of eight anti-pp65 positive sera, only one strongly and another weakly react to HeLa antigens at 65 kD position.

### Induction of anti-nuclear antibody (ANA) by pp65_336-439_-immunization

**Figure 3:**
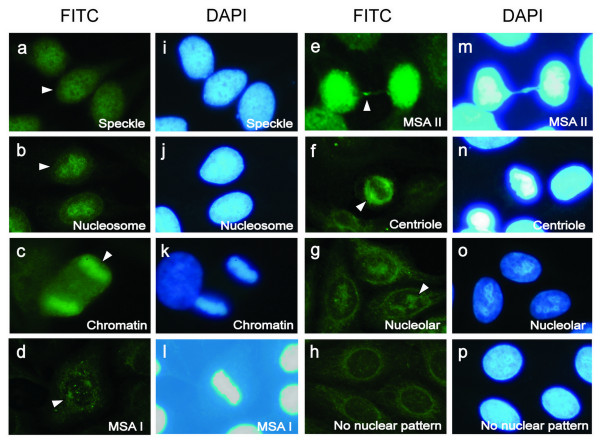
**Detection of anti-nuclear reactivity in pp65_1-167_, pp65_336-439_, SA-C3d or PBS immunized sera**. HEp-2 substrate slides were used for detection of anti-nuclear antibodies. Sera from eight weeks post-immunization were 100X diluted for ANA stains. Patterns of speckle (**a, i**), chromatin (**b, j**), nucleosome (**c, k**), MSA I (**d, l**), MSA II (**e, m**) and centriole (**f, n**) were revealed with sera from pp65_336-439_-immunized mice. Nucleolar response (**g, o**) was detected in sera from pp65_1-167 _or pp65_336-439 _immunized mice. Nuclear reactivity was not found in SA-C3d or PBS immunized mice (**h, p**). White arrowheads indicate the pattern of nuclear responses. MSA I/II: mitotic spindle type I/II.

To determine if pp65_336-439 _immunization could induce antibodies against nuclear components from HeLa cells, the anti-nuclear antibody (ANA) test was performed. The results showed that pp65_336-439 _immunization induced multiple ANA staining patterns, including speckled (5/17, Figure 3a, i), nucleosome (4/17, Figure 3b, j), chromatin (4/17, Figure 3c, k), mitotic spindle type I (MSA I, 4/17, Figure 3d, l), mitotic spindle type II (MSA II, 10/17, Figure 3e, m) centriole (6/17, Figure 3f, n) and nucleolar (14/17, Figure 3g, o) stains at 1:100 dilution at 8 weeks and continued to 12 weeks post-immunization. In several occasions, ANA patterns were detected at dilution as much as 500-fold. Nuclear stains, however, were not detected from either SA-C3d or PBS-immunized animals (0/5, 0/2, Figure 3h, p). Four pp65_1-167_-immunized mice developed weak anti-nucleolar reactivity (4/11, Figure 3g, o) detectable at 1:40 dilution. Taken together, pp65_336-439 _immunization could induce cross-reactive antibodies to multiple nucleus components (Table 4).

**Table 4:** The summary of ANA patterns in mice against to cellular components

Nuclear patterns	pp65_1-167_ *n *= 11	pp65_336-439_ *n *= 17	SA-C3d *n *= 5	PBS *n *= 2
Speckle	0	5	0	0
Nucleosome	0	4	0	0
Chromatin	0	4	0	0
Centriole	0	6	0	0
MSA I	0	4	0	0
MSA II	0	10	0	0
Nucleolar	4	14	0	0

MSA I, mitotic spindle type I, MSA II, mitotic spindle type II

### Induction of anti-dsDNA antibody by pp65_336-439 _immunization

**Figure 4:**
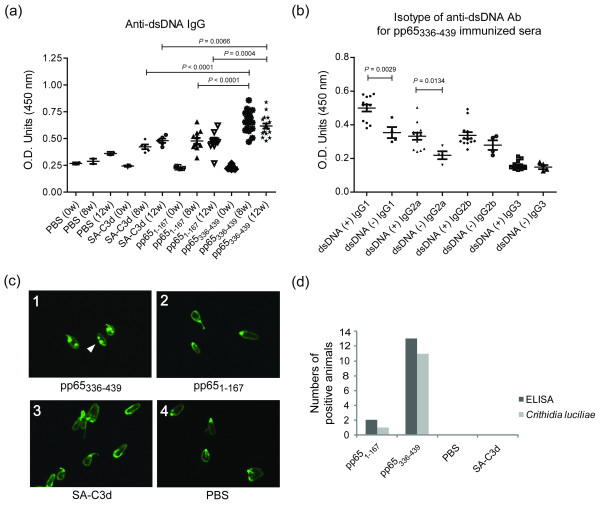
**Detection of anti-dsDNA antibody in pp65_1-167_, pp65_336-439_, SA-C3d or PBS immunized sera**. (**a**) ELISA assays for anti-dsDNA activity at 1:100 serum dilution. The dsDNA positivity is defined by mean + 3 s.e.m. of SA-C3d-immunized sera. O.D._450 _> 0.49 was considered to be positive. (**b**) Isotyping of anti-dsDNA antibody for pp65_336-439_-immunized mice (*n *= 17) at eight weeks post-immunization. Tests were performed on ELISA at 1:100 dilution. dsDNA(+): dsDNA seropositive, dsDNA(-): dsDNA seronegative. Graphs depict mean ± s.e.m. values. Unpaired Student *t *test was performed. Results with a *P-*value of < 0.05 were considered to be significant. (**c**) Representations of *Crithidia luciliae *stain by sera from (**c1**) pp65_336-439_, (**c2**) pp65_1-167_, (**c3**) SA-C3d or (**c4**) PBS immunized animals at eight weeks post-immunization at 1:40 dilution. White arrowheads indicate dsDNA positive stains. (**d**) The summarized results of ELISA assays and *Crithidia luciliae *stains. These results are representative of triplicated experiments.

Anti-dsDNA antibody is a feature and a disease indicator for SLE patients [20-22]. ELISA assays showed that pp65_336-439_-immunized sera exhibited significantly enhanced anti-dsDNA antibody activity compared to animals immunized with pp65_1-167 _(pp65_336-439 _vs. pp65_1-167_, 0.66 ± 0.02 vs. 0.48 ± 0.03, *P *< 0.0001), or SA-C3d (pp65_336-439 _vs. SA-C3d, 0.66 ± 0.02 vs.0.42 ± 0.02, *P *< 0.0001) at 8 weeks and continued to 12 weeks post-immunization (Figure 4a). The differences of anti-dsDNA antibody between pp65_1-167 _and SA-C3d immunized mice were insignificant. The IgG2a to dsDNA is the dominant isotype to SLE nephritis [23]. ELISA-based assays showed that 13 of 17 pp65_336-439 _immunized mice were positive to dsDNA. Isotyping showed that enhanced IgG1 (dsDNA (+) IgG1 vs. dsDNA (-) IgG1, 0.50 ± 0.02 vs. 0.35 ± 0.03, *P *= 0.0029) and IgG2a isotypes (dsDNA (+) IgG2a vs. dsDNA (-) IgG2a, 0.33 ± 0.02 vs. 0.22 ± 0.02, *P *= 0.0134) were the contributors of anti-dsDNA activity (Figure 4b). To confirm the ELISA-based anti-dsDNA analysis, the *Crithidia luciliae *stains were performed. Of 17 pp65_336-439_-immunized animals, 11 were positive for anti-dsDNA antibody (1:40 dilution) at 8 weeks and continued to 12 weeks post-immunization, compared to 2 of 11 pp65_1-167_-immunized mice (Figure 4c). All *Crithidia luciliae*-positive sera were positive at ELISA tests. Only one pp65_1-167_-immunized mouse was positive for *Crithidia luciliae *at 12 weeks post-immunization (Figure 4d).

### The elevated anti-HCMV pp65_336-439 _antibody is cross-reactive to dsDNA and nucleus components

**Figure 5:**
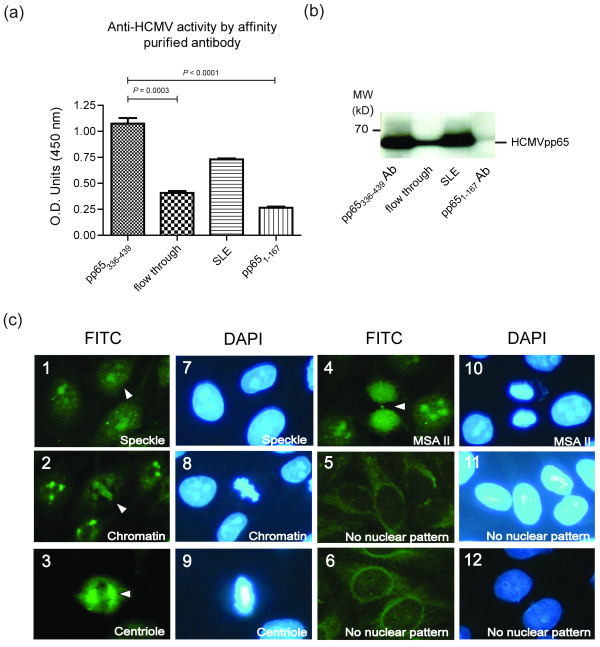
**Detection of anti-nuclear reactivity in pp65_1-167 _or pp65_336-439 _specific antibodies from eight weeks post-immunization sera**. **(a) **ELISA assays of affinity purified anti-pp65_336-439 _antibody (pp65_336-439_), anti-pp65_1-167 _antibody (pp65_1-167_), SLE patients' sera (SLE) and flow through against purified HCMV virions. Graphs depict mean ± s.e.m. values. Unpaired Student *t *test was performed. Results with a *P-*value of < 0.05 were considered to be significant. **(b) **Immunoblot assays on affinity purified anti-pp65_336-439 _antibody (pp65_336-439_), anti-pp65_1-167 _antibody (pp65_1-167_), SLE patients' sera (SLE) and flow through against purified HCMV virions. Molecular mass markers (kD) are shown on the left. MW: molecular weight. **(c) **ANA stains with HEp-2 substrate slides were performed with HCMV affinity-purified antibody. Patterns of speckle **(c_1_, c_7_)**, chromatin **(c_2_, c_8_)**, centriole **(c_3_, c_9_) **and MSA II **(c_4_, c_10_) **were revealed with affinity purified anti-pp65_336-439 _antibodies. Nuclear pattern was not found in flow through **(c_5_, c_11_) **or affinity-purified anti-pp65_1-167 _antibody **(c_6_, c_12_) **stains. White arrowheads indicate the patterns of nuclear response. MSA II: mitotic spindle type II.

**Figure 6:**
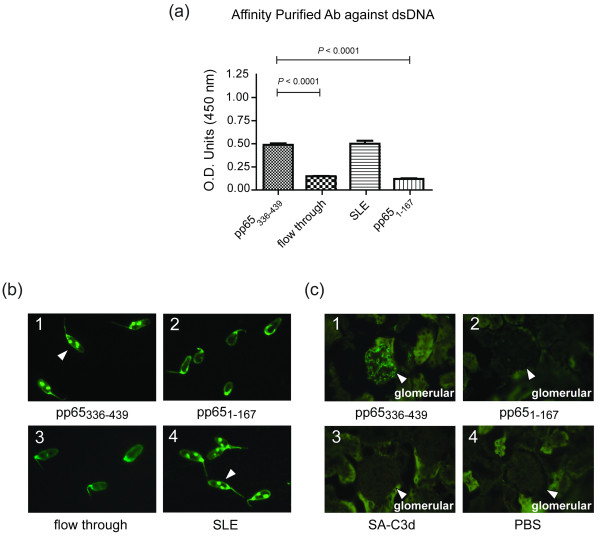
**The cross-reactivity of purified anti-pp65_336-439 _or anti-pp65_1-167 _antibody to dsDNA**. **(a) **ELISA assays for affinity-purified anti-pp65_336-439 _antibody (pp65_336-439_), anti-pp65_1-167 _antibody (pp65_1-167_), flow through and SLE patients' sera (SLE) against dsDNA. Graphs depict mean ± s.e.m. values. Unpaired Student *t *test was performed. Results with a *P-*value of < 0.05 were considered to be significant. **(b) **Representations of *Crithidia luciliae *stain by affinity-purified anti-pp65_336-439 _antibody (**b_1_**, pp65_336-439_), anti-pp65_1-167 _antibody (**b_2_**, pp65_1-167_), flow through **(b_3_) **and SLE patients' sera (**b_4_**, SLE) against dsDNA. White arrowheads indicate the positive stains. **(c) **Representations of Immunofluorescent stain for immunoglobulin deposition on glomerular by pp65_336-439 _**(c_1_) **immunized mice, pp65_1-167 _**(c_2_) **immunized mice, SA-C3d **(c_3_) **immunized mice or PBS **(c_4_) **controls. Kidneys were collected at 20 weeks of age. White arrowheads indicate antibody deposition on glomerular. dsDNA: double-stranded DNA.

To elucidate the relation between pp65_336-439 _immunization and anti-nuclear antibody found in animals, antibodies to either pp65_336-439 _or pp65_1-167 _were affinity purified from pooled pp65_336-439 _or pp65_1-167 _immunized mouse sera. The results showed that affinity-purified pp65_336-439_-specific IgG exhibiting significantly enhanced anti-HCMV activity compare to pp65_1-167 _specific IgG (pp65_336-439 _vs. pp65_1-167_, 1.08 ± 0.05 vs. 0.27 ± 0.01, *P *< 0.0001, Figure 5a). Unbound fractions (flow through) from purification processes remain anti-HCMV positive, but the titer reduced significantly (pp65_336-439 _vs. flow through, 1.08 ± 0.05 vs. 0.41 ± 0.02, *P *= 0.0003, Figure 5a, b). As immunofluorescent stains performed in Figure 3, affinity-purified anti-pp65_336-439 _antibodies reproduced all ANA stains found in direct serum-staining (Figure 3), including speckled (Figure 5c1,c7), chromatin (Figure 5c2,c8), centriole (Figure 5c3,c9) or MSA II (Figure 5c4,c10) stains. Antibodies purified from flow through or anti-pp65_1-167 _immunized sera, however, did not produce noticeable nuclear staining patterns (Figure 5c5,c11 and Figure 5c6,c12). In addition to nuclear stain, affinity-purified anti-pp65_336-439 _antibody also possessed reactivity to dsDNA as ELISA and *Crithidia luciliae *slides demonstrated (0.49 ± 0.02, Figure 6a, b). The difference of anti-dsDNA activity between purified anti-pp65_1-167 _antibody and flow through were insignificant (pp65_1-167 _vs. flow through, 0.12 ± 0.01 vs. 0.15 ± 0.01).

**Figure 7:**
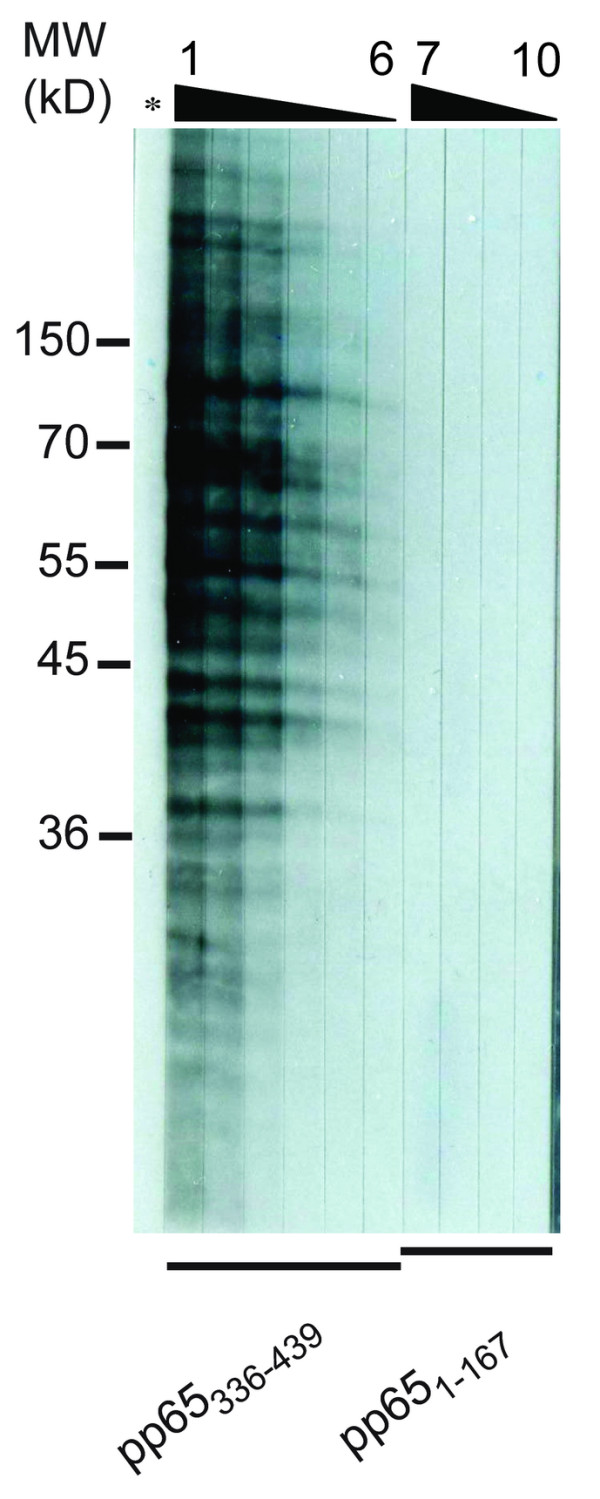
**Detection of protein-to-protein interaction between pp65_336-439 _and HeLa lysate**. Whole HeLa lysate was separated by SDS-PAGE and transferred to nitrocellulose paper. The pp65_336-439 _or pp65_1-167 _His-tag fusion peptide was incubated with blot at concentrations of 20, 10, 5, 2.5, 1.25, 0.625 mg/ml (No.1 to 6) or at concentrations of 20, 10, 5, 2.5 mg/ml (No.7 to 10), respectively. Asterisk indicates protein-free negative control. Molecular mass markers (kD) are shown on the left. MW: molecular weight (kD).

Deposition of immunoglobulin or immune complexes on glomeruli is a characteristic of early nephritis and is often found in SLE patients [24]. Immunofluorescent stains with anti-mouse IgG in renal section showed that pp65_336-439_-immunized mice developed signs of deposition of immune complex on glomeruli (Figure 6c1). A total of 6 of 17 pp65_336-439_-immunized mice showed IgG deposition on glomeruli, but such stains were not found in pp65_1-167 _(0/11, Figure 6c2), SA-C3d (0/5, Figure 6c3) or PBS (0/2, Figure 6c4) treated animals. We did not observe clinical symptoms, such as proteinuria or lesions on the kidneys of immunofluorescent-positive mice. Nevertheless, it is noteworthy that the levels of immunoglobulin deposition showed positive correlation to the titers of the anti-dsDNA antibody (data not shown).

### Yeast two-hybrid showed the binding of HCMVpp65_336-439 _binds to diverse HeLa proteins

Immediately after infection, HCMVpp65 was transported into the nucleus and migrated to nucleolus [25]. We hypothesized that the pp65_336-439 _fragment may complex to intracellular antigens during infection. Immunoblotting and yeast two-hybrid verified this hypothesis and showed the association between HeLa proteins and pp65_336-439 _(Figure 7, Table 5). Such binding was not found on either pp65_1-167 _or protein-free tests. Among those pp65_336-439 _binding colonies from yeast two-hybrid, 10 were verified as nucleic acid binding proteins, heat-shock proteins and apoptosis-related proteins (Table 5).

**Table 5:** List of pp65_336-439 _binding proteins from yeast two-hybrid system

Clones	Binding proteins
Nuclear proteins	
AD80	Thyroid hormone receptor associated protein 3
AD107	Ribosomal RNA processing 8
AD99	CAF1A chromatin assembly factor 1, subunit A
AD127	Protein DBF4 homolog A
Apoptotic related proteins	
AD135	Transcriptional regulator protein (HCNGP, SAP30 BP)
AD140	YSK4 Sps1/Ste20-related kinase
AD365	Dermal papilla-derived protein 6 isoform 3
AD22	Cytochrome c oxidase subunit III
Heat shock associated protein	
AD10	DnaJB3 (Hsp40)
AD87	DnaJB8 (Hsp40)

## Discussion

HCMVpp65 is a strong T-cell antigen to most human and several epitopes within this antigen have been mapped from normal population [14,26]. Up to now, 28 CTL epitopes have been reported for pp65 and four of them are located within pp65_336-439 _[14]. The elevation of anti-pp65 antibody is not a rare phenomenon to persons during primary HCMV infection or reactivation, but such activity is rarely sustained [27]. In this study, we reported at least one SLE-specific, autoimmune-prone B-cell epitope within HCMVpp65_336-439_. B-cell epitope(s) within this region may not be unique to SLE sera, since our mapping has not ruled out the possible existence of epitope(s) within the junctions of fragments or conformational epitope(s). Elevated and sustained anti-pp65 antibody in SLE patients and induction of autoimmunity via immunization in both of BALB/c and NZB/W mice suggesting a hypothesis that humoral immunity toward pp65 antigen may possess a pathogenic potential [15].

Transient immunity to pp65 can be detected in the sera of BALB/c mice immunized with either full-length pp65 or pp65_336-439 _in Freund's adjuvant [15]. To study the effect of anti-pp65 antibody, we have immunized BALB/c mice with either full-length or truncated pp65 peptides [15]. In addition, a potent adjuvant that is capable of eliciting strong and sustained immunity to an antigen of low immunogenicity, such as pp65, is preferred. The C3d is an effective molecular adjuvant that appears safe and acceptable for use in vaccines [17]. Dempsey *et al. *showed that C3d-conjugated hen egg lysozyme (HEL) exhibited up to a 1,000-fold increase in immunogenicity than HEL alone [16]. We found that immunization of pp65_336-439 _peptide with C3d could sustain the humoral immunity to pp65_336-439_, and such immunization also elicited cross-reactive antibody against host cellular proteins, including dsDNA and its associated proteins on non-autoimmune BALB/c mice.

The etiology of autoimmunity is complex, either the cross-reactivity by anti-pp65_336-439 _antibody to multiple nuclear components or epitope spreading by binding of pp65 to host proteins may contribute to the out spread of auto-reactive antibodies. We could not identify significant sequence homology between pp65_336-439 _and many nuclear targets recognized by SLE sera. Diamond *et al. *showed that decapeptide DWEYSVWLSN could induce anti-dsDNA IgG and deposition of immunoglobulin on glomeruli [28].

McClain *et al. *reported that the immunization with either EBNA-1 could induced specific anti-Ro and anti-EBNA-1_52-72 _antibody in experimental animal, but EBNA-1_52-72_, with this structure, shared no amino acid sequence homology with Ro [3]. Sunder *et al. *revealed that immunization of EBNA-1 not only induced anti-EBNA-1 antibody in mice, but also exhibited cross-reactive antibodies to both SmB/B' and dsDNA [29]. These studies suggested the similarity of amino acid sequence is not a requirement for mimicry-induced autoimmunity.

Our BALB/c mice developed sustained antibodies to pp65_336-439 _and nuclear components following immunization. Our immunization scheme not only sustained the anti-pp65 activity, but also induced high titers of antibodies to nuclear components, including the nucleosome, centriole and chromatin. Such weak anti-nuclear responses were found on few animals never exposed to pp65_336-439_. This weak anti-nuclear activity is likely due to adjuvant-induced polyclonal activation because neither ANA activity nor the *Crithidia luciliae *stain was found from affinity-purified anti-pp65_1-167 _antibody. Cross-reactivity between a foreign antigen and an autoantigen is a characteristic of mimicry-induced autoimmunity [30]. Our affinity purification results demonstrated that the anti-pp65_336-439 _antibodies cross-react to several nuclear antigens, including dsDNA, suggesting that mimicry could play a part in the pp65-induced tolerance break. The anti-dsDNA antibody, particularly IgG2a, was reported to SLE nephritis and also identified from our pp65_336-439_-immunized BALB/c [31,32]. Nevertheless, the direct relation to nephritis by anti-pp65_336-439 _initiated anti-dsDNA antibody of IgG2 isotype was not studied in this work. The anti-dsDNA antibody from pp65_336-439_-immunized mice was detected as early as four weeks, suggesting that pp65_336-439 _is a potent inductor of cross-reactivity. The *Crithidia luciliae *stain has been the golden standard to anti-dsDNA antibody. Of 17 pp65_336-439_-immunized mice, 11 and 13 mice were positive for the *Crithidia luciliae *stains and ELISA assays, respectively. All *Crithidia luciliae*-positive mouse sera also possessed high titers of anti-dsDNA activity by ELISA assays, confirming the significant elevated anti-dsDNA reactivity in pp65_336-439_-immunized animals.

The peptide-induced immunity, which cross-reacts with both dsDNA and α-actinin, has been described and its pathogenesis was illustrated [33]. Similar to anti-DWEYSVWLSN antibody, affinity-purified anti-pp65_336-439 _antibody from pp65_336-439_-immunized animals cross-reacted with dsDNA on both *Crithidia luciliae *stains and ELISA assays. Such anti-dsDNA reactivity was not found in pp65_1-167 _or adjuvant immunized animals. To the best of our knowledge, HCMV has not been reported to induce the anti-dsDNA antibody. The pathogenicity of pp65_336-439_-induced anti-dsDNA antibody on BALB/c animals was not fully examined by this study. Nevertheless, we found precipitation of immune complexes on glomeruli at 12 weeks post-immunization (20 weeks of age), and noticed positive correlation of anti-dsDNA titers to the complex precipitation (data not shown). This finding implies that an early stage of renal pathogenesis that resembles SLE nephritis may have been induced by pp65_336-439_-mediated cross-reactive antibody. Arbuckle *et al. *have revealed that anti-dsDNA antibody could be found as early as nine years before the diagnosis of SLE [22]. The asymptomatic existence of anti-dsDNA activity in our animals suggests an extended observation is required to demonstrate the clinical consequences by pp65 immunization. Genetics plays an essential role on pathogenesis of autoimmunity that might also explain the lack of clinical symptoms on our animals following immunization [34].

In addition to mimicry, epitope spreading could be another driving force to pp65_336-439_-induced autoimmunity. The T-antigen of human polyomaviruses has been shown to complex with nucleosomes of infected cells during viral replication. These nucleosomes/T-antigen complexes are subsequently targeted by immune responses and become a catalyst for cross-reactive antibodies against both virus and host [35]. At HCMV infection, pp65 is imported to the nucleus immediately via two nuclear localization sequences: pp65_418-438 _and pp65_537-561 _[36]. The pp65 has been demonstrated to bind to metaphase-arrested chromosomes in the pp65-expressing fibroblasts during productive virus infection [37]. These findings prompted us to hypothesize that pp65 may not only bind to cellular proteins, but also form immune-complexes to DNA or other nuclear components. As expected, pp65_336-439 _bound multiple cellular proteins including nucleic acid binding proteins, nuclear proteins, apoptosis-related proteins and heat-shock proteins (Table 5). It is noteworthy that nuclear proteins and nucleic acid binding proteins are common targets to autoimmunity, and abnormal apoptosis has been associated with autoimmunity [38]. These findings suggest that antigen-bound cellular proteins shared high probability of becoming immunogenic and provide a mechanism for subsequent development of autoimmunity. Therefore, binding to intracellular proteins by full-length or fragmented pp65 may not only generate immune-complexes (virus/host) that are subsequently targeted by antiviral antibodies but also increase the chance of epitope spreading and lead to autoimmunity in persons with susceptible genetic backgrounds.

## Conclusions

The antibody against HCMVpp65_380-439 _antibody is rare in healthy populations but is a common feature among SLE sera. Through immunization of pp65_336-439 _with C3d as adjuvant, we were able to sustain the antibody titers to pp65_336-439 _peptide and demonstrate cross-reactivity of anti-pp65 antibody to nuclear components, including dsDNA on BALB/c mice. Yeast two-hybrid analysis revealed that pp65_336-439 _could bind to nuclear proteins, suggesting the immune-complexes of pp65 and nuclear proteins may be part of the trigger to autoimmunity. Although none of the experimental animals developed SLE-like clinical symptom, deposition of immunoglobulin was identified from pp65_336-439_-immunized animals at 12 weeks post-immunization. Therefore, a sustained humoral immunity to pp65 may present a risk to individuals with a background predisposed to SLE.

## Abbreviations

ANAs: anti-nuclear antibodies; C3d: complement 3d; CFA: complete Freund's adjuvant; CnBr: cyanogen bromide; CTD: connective tissue disease; dsDNA: double-stranded DNA; EBNA-1: Epstein-Barr virus nuclear antigen 1; EBV: Epstein-Barr virus; ELISA: Enzyme Linked Immunosorbent Assay; FITC: fluorescein isothiocyanate; HCMV: human cytomegalovirus; HEL: hen egg lysozyme; HRP: horseradish peroxidase; IFA: incomplete Freund's adjuvant; MSA-I/II: mitotic spindle type I/II; OPD: o-phenylenediamine dihydrochloride; PBS: phosphate-buffered saline; pp65: phosphoprotein 65; RA: rheumatoid arthritis; RT: room temperature; SA: streptavidin; SLE: systemic lupus erythematosus; SmB/B': Smith antigen B/B'; snRNP: small nuclear ribonucleoprotein; SS: Sjögren's syndrome; SSc: systemic sclerosis; YNB: yeast nitrogen base.

## Competing interests

The authors declare that they have no competing interests.

## Authors' contributions

MC, SLW and AHH jointly contributed to the design of the study. AHH performed ELISA, Western blot and immunofluorescence. YJJ carried out the yeast two-hybrid. CTL participated in the tissue stains. AHH, MC and SLW were responsible for data analysis and interpretations. AHH and MC wrote the manuscript. All authors read and approved the final manuscript.
